# Insights into organoid-based modeling of COVID-19 pathology

**DOI:** 10.1186/s12985-023-01996-2

**Published:** 2023-02-25

**Authors:** Mohadese Hashem Boroojerdi, Tariq Al Jabry, Seyed Mohamad Javad Mirarefin, Halima Albalushi

**Affiliations:** 1 Department of Human and Clinical Anatomy, College of Medicine and Health Sciences, Sultan Qaboos University, Muscat, Oman; 2 Department of Genetics, College of Medicine and Health Sciences, Sultan Qaboos University, Muscat, Oman; 3 Department of Immunology, Faculty of Medical Sciences, Tarbiat Modares University, Tehran, Iran

**Keywords:** COVID-19, SARS-CoV-2, Organoid, Stem cell, Regenerative medicine

## Abstract

Since December 2019, various types of strategies have been applied due to the emergent need to investigate the biology and pathogenesis of severe acute respiratory syndrome coronavirus 2 (SARS-CoV-2) to discover a functional treatment. Different disease modeling systems, such as mini-organ technology, have been used to improve our understanding of SARS-CoV-2 physiology and pathology. During the past 2 years, regenerative medicine research has shown the supportive role of organoid modeling in controlling coronavirus disease 2019 (COVID-19) through optimal drug and therapeutic approach improvement. Here, we overview some efforts that have been made to study SARS-CoV-2 by mimicking COVID-19 using stem cells. In addition, we summarize a perspective of drug development in COVID-19 treatment via organoid-based studies.

## Introduction

The initial account of an acute respiratory syndrome caused by an unknown virus was reported in December 2019 in China [1, 2]. This condition was later known as coronavirus disease-2019 (COVID-19), which was found to be caused by severe acute respiratory syndrome coronavirus 2 (SARS-CoV-2) [2]. COVID-19 is characterized by respiratory defect signs with the ability to spread to other organs. It has been found to be associated with severe risks in elderly and immunocompromised individuals [3].

Many efforts have been made to establish feasible approaches to manage COVID-19. The utilization of an appropriate model system has been central to this approach, where promising strategies to investigate the pathogenesis of SARS-CoV-2 and the development of proper treatment modalities are underway [4, 5]. Since investigating the pathogenesis of a disease in human beings is not always possible, model systems can be employed to manage this restriction. One of the best examples in this regard is the use of stem cell-based patterns, animal models, and organoids [6]. Each technique has its own limitations; for instance, in vivo models are very expensive to create. On the other hand, cell-based methods require the application of primary cells or cell lines that are not easily available. Additionally, cell-based models fail to model exactly what happens in a human body [7, 8]. Recently, ‘organoids’ have proven to be effective disease models that could remarkably mimic the human body environment to study virus pathogenesis. Organoids are three-dimensional cellular organizations that can mimic the structures and functions of tissues of interest. They can be derived from pluripotent stem cells (PSCs), such as induced pluripotent stem cells (iPSCs), embryonic stem cells (ESCs), or adult stem cells (ASCs). In addition, organoids are cheaper than animal models and do not have the ethical limitations of animal models. Recently, several researchers have generated organoids to investigate the pathophysiology of SARS-CoV-2 and to determine optimal drug candidates for the treatment of COVID-19 [9].

### Current target-based approaches for COVID-19 treatment

At the beginning of the SARS-CoV-2 outbreak, researchers tried to design experiments to study the pathology of the virus and to develop possible treatment strategies based on their previous knowledge of other coronavirus subtypes. Several types of existing approved medications were used to control COVID-19, and fortunately, some of them showed promising outcomes [10]. Treatment strategies based on targeting viral entry biomarkers, in an attempt to block viral entry into host cells, are considered promising approaches for managing COVID-19. Based on previous studies, angiotensin-converting enzyme 2 (ACE2), transmembrane protease serine subfamily 2 (TMPRSS2), and CD147 were reported to be important human cellular receptors necessary for SARS-CoV-2 entry into host cells. These markers are expressed by various types of cells in the human body and could justify the spread of SARS-CoV-2 infection to certain organs.

#### ACE2 blockade

ACE2 is the most significant entry point for SARS-CoV-2. Several medications have been found to be practical ACE2 inhibitors, such as benazepril, lotensin, captopril, eralapril, and Vasotec. A high expression level of ACE2 receptors has been detected in the epithelial cells of the respiratory system, making them a primary site of SARS-CoV-2 infection. In addition, the high expression level of ACE2 in the gastrointestinal system epithelium makes these cells the second target for SARS-CoV-2 infection [11, 12]. RNA expression levels have also suggested that other organs, such as the brain, kidney, liver, and eye, significantly express ACE2 [13–20].

#### TMPRSS2 inhibitors

The SARS-CoV-2 spike (S) protein binds to TMPRSS2, which is expressed by epithelial cells in different organs and contributes to the viral entry procedure [21, 22]. As a result, applying a TMPRSS2 inhibitor was suggested as a favorable therapeutic approach [23]. In recent years, several inhibitors have been introduced to block TMPRSS2. Nafamostat (a synthetic serine protease inhibitor) and camostat (a protease inhibitor) are the most commonly used inhibitors to block SARS-CoV-2 fusion to the host cell membrane [17, 24]. Moreover, combination therapy utilizing neutralizing antibodies together with TMPRSS2 inhibitors has also been suggested as a useful method for COVID-19 treatment [23, 25].

#### Inhibitors targeting CD147

CD147 is another important molecule that plays a significant role in SARS-CoV-2 entry into host cells through direct interactions with the SARS-CoV-2 spike protein. Azithromycin is a known antibiotic with anti-inflammatory properties that could possibly inhibit CD147 [26]. The anti-inflammatory characteristics of this antibiotic could control the severity of COVID-19 in the early stages of the disease via cytokine level reduction. It is also affordable and widely available, which makes it an appropriate candidate for COVID-19 management [26, 27]. Another CD147 inhibitor is meplazumab, which is a monoclonal antibody that targets the CD147 receptor effectively and blocks SARS-CoV-2 access to host cells expressing CD147. Meplazumab may assist in the management of post-COVID-19 cytokine storm syndrome via its proposed influence on proinflammatory cytokines [26, 28].

### Cell-based therapy for COVID-19 treatment

Cell-based therapy, especially with mesenchymal stem cells (MSCs), is an emerging strategy in COVID-19 therapy. MSCs are multipotent stromal cells with the capacity for self-renewal and differentiation into various types of cells. They demonstrate immunomodulatory properties through the production of different types of cytokines [29]. The anti-inflammatory activities of MSCs make them capable of controlling acute respiratory distress syndrome (ARDS) and lung damage. As a result, MSCs have been suggested as a suitable candidate for improving clinical outcomes in COVID-19 patients. Over 80 clinical trials have investigated the clinical effects of MSC-based therapy on patients with COVID-19 as of January 16, 2022 [6, 30, 31].

### Organoid systems for studying SARS-CoV-2 pathogenies

Since December 2019, approximately 472 million patients have been diagnosed with COVID-19, and approximately six million deaths have been reported globally [32]. Since the primary infection caused by SARS-CoV-2 is respiratory-based, developing a lung organoid model might be a promising approach for studying SARS-CoV-2 pathogenesis and drug screening. Therefore, a high number of studies have focused on transformed cell lines that mimic the human lung environment [32].

#### Respiratory system organoids

**Fig. 1:**
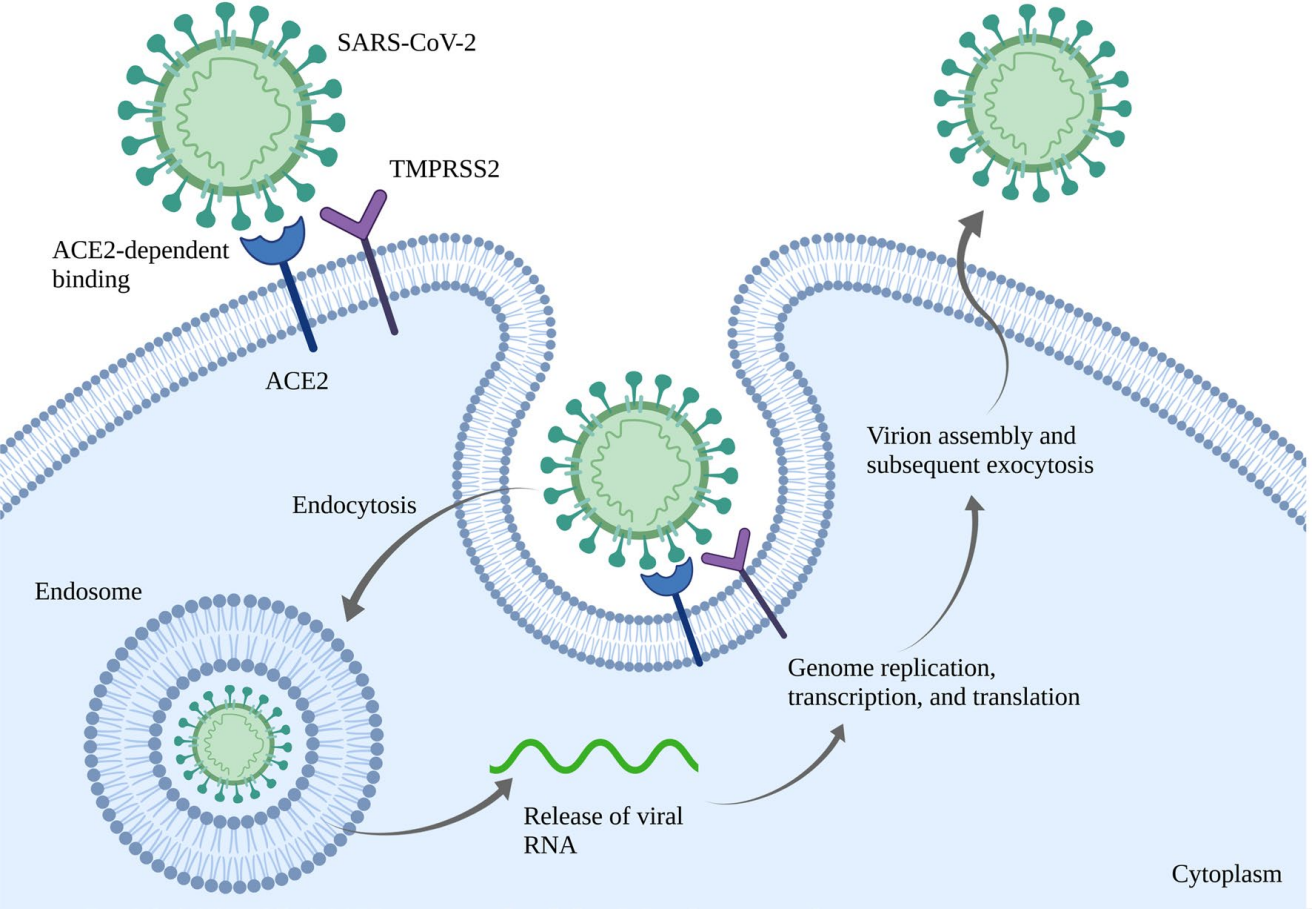
The process of ACE2 and TMPRSS2-assisted SARS-CoV-2 internalization

As mentioned before, human cellular receptors such as ACE2 and TMPRSS2 are the most important internalization points for SARS-CoV-2. Expression of ACE2 by alveolar cells in alveoli and lung mucosa make these cells suitable candidates for SARS-CoV-2 internalization. In fact, one of the reasons for lung damage following SARS-CoV-2 infection is the reduction in alveolar cells [33]. Furthermore, SARS-CoV-2 infection can cause ARDS via ACE2 downregulation. It can also remarkably impair oxygen transportation in the blood circulation [16]. The schematic process of SARS-CoV-2 internalization assisted by ACE2 and TMPRSS2 is shown in Fig. 1.

Lung organoids have recently been utilized to study SARS-CoV-2 pathogenesis and for drug screening. Han et al. (2020) used human pluripotent stem cells (hPSCs) to form lung organoids and demonstrated through RNA analysis a high level of cytokine and chemokine enhancement but low interferon type I/III signaling following SARS-CoV-2 infection. Another study demonstrated that ACE2 expression in lung organoids makes them in a passive state to SARS-CoV-2 infection. The same study reported that treating the infected hPSC-derived lung organoids with SARS-CoV-2 internalization inhibitors, such as imatinib and mycophenolic acid (also known as MPA), caused a reduction in SARS-CoV-2 infection in these organoids [32]. SARS-CoV-2 was also reported to show the potential to infect the bronchi alveolar organoid systems through alveolar type II-like cells [34]. These findings were consistent with what happens in the bronchi of COVID-19 patients. With regard to COVID-19, interferons can limit SARS-COV-2 infection severity and/or cause higher disease severity [34]. In fact, the severity of manifestations in COVID-19 patients varies from one person to another. Notably, ACE2 expression levels can be enhanced through inflammatory signals by interferon production [7]. In line with these findings, Hou et al. (2020) reported that healthy individuals exhibited the lowest expression level of ACE2. A high level of expression of ACE2 was found in ciliated cells of the respiratory tract (especially in the proximal part of the airway) (Hikmet et al., 2020; Hou et al., 2020), suggesting that ciliated cells may be more prone to infection than alveolar cells. Altogether, these studies demonstrated that alveolar cells could not be considered the first candidate for SARS-CoV-2 infection [7, 35, 36]. Together, these studies presented airway organoids as versatile models for the in vitro study of infectious pulmonary diseases [7].

To improve COVID-19 drug discovery, Tatsuya et al. (2021) generated human bronchial mini-organs from human bronchial epithelial cells that are commercially accessible. These cells expressed high levels of ACE and TMPRSS2 receptors. Following SARS-CoV-2 infection, the gene expression profiling of infected organoids was performed via RNA-seq analysis, and the results demonstrated reasonable interferon type I signal enhancement. In addition, the results also revealed the potential of camostat as a limiter of SARS-CoV-2 replication. Camostat controls COVID-19 disease by inhibiting the TMPRSS receptor [34].

With regard to drug screening improvements to inhibit SARS-CoV-2 internalization, Suzuki et al. (2020) generated lung organoids from human iPSCs along with alveolar epithelial cells with high expression of ACE2 and TMPRSS receptors. The expression of these receptors by the mentioned cells could make them permissive to SARS-CoV-2 infection. Suzuki et al. (2020) reported that lung organoid infection by SARS-CoV-2 activates genes such as IL-18 and Caspase-1, which are responsible for the inflammatory response. The activation of these inflammatory pathways causes lung cell inflammation and can subsequently lead to death. In addition, tumor necrosis factor-alpha (TNF-alpha), IL-6, IL-8, and IFN expression and production enhancement following SARS-COV-2 infection can lead to a cytokine storm and subsequently result in serious lung injury [10]. Similarly, Hoffmann et al. (2020) reported the inhibition of spike protein-mediated SARS-CoV-2 internalization following TMPRSS2 inhibition by camostat and nafamostat [11].

Salahudeen et al. (2020) created lung organoids with the potential to express ACE2 on their external surface to simplify Type II alveolar epithelial cell (AT2) infection by SARS-CoV-2. The structure of the organoids derived from human AT2 cells provided a proper model to detect the SARS-CoV-2 target cell population. According to the results of this study, single-cell analysis of cells that created the organoids demonstrated diversity in their function [37, 38].

COVID-19 patients exhibit a wide range of clinical symptoms. Hou et al. (2020) used a reverse genetic system to engineer SARS-CoV-2 with green fluorescent protein (GFP) to study the pathogenesis of SARS-CoV-2. They demonstrated the highest expression of ACE2 in the nose through high-resolution in situ hybridization. They also reported that this expression is reduced in the lower parts of the respiratory tract. As a result, the severity of SARS-CoV-2 decreased throughout the proximal to the distal respiratory system. The results of this study highlighted the vulnerability of the nasal cavity to SARS-CoV-2 [7].

A very significant characteristic of the SARS-CoV-2 spike protein is the presence of a multibasic cleavage site. The spike protein has the main role in cell–cell adhesion and SARS-CoV-2 entry in COVID-19. Mykytyn et al. (2021) found that the multibasic cleavage site in the SARS-CoV-2 spike protein enhances the virus infectivity potential in human airway mini-organs. Additionally, the multibasic cleavage site plays a significant role in the enhancement of SARS-CoV-2 entry speed into the cells via TMSPRSS. They confirm that by showing the effect of TMSPRSS2 blockade in controlling SARS-CoV-2 entry to the human airway mini-organs. Their results suggest that TMSPRSS2 is the main entry route for SARS-CoV-2 in lung organoids, which makes it a practical target for therapeutic use [39].

In the past, the most common method for virus production was using transformed cell lines. This method could lead to the adaptation of the virus to the cell culture environment through high viral mutation rates. For example, a multibasic cleavage site mutation or deletion in the spike protein could take place. Recently, it has been reported that human airway cells prevent SARS-CoV-2 multibasic cleavage site cell culture adaptation. Previously, Mykytyn et al. (2021) indicated the role of the multibasic cleavage site in facilitating SARS-CoV-2 entry into airway cells through TMSPRSS [39]. Moreover, Lamers et al. (2021) indicated that SARS-CoV-2 propagation in human airway organoids with an active TMPRSS2-mediated entry pathway could stop adaptations of cell culture in the multibasic cleavage site. Alternatively, using organoid models could be a suitable approach for SARS-CoV-2 replication without the risk of adaptation to culture conditions [34].

#### Brain organoids

Evidence of SARS-CoV-2 brain infections is scarce [40]. However, SARS-CoV-2 RNA has been detected in the cerebrospinal fluid (CSF) of COVID-19 patients [41], and various neurological symptoms have also been documented [42–44]. COVID-19 has been implicated in a variety of neurological symptoms, including headache, seizure, confusion, dizziness, hyposmia (loss of smell), hypogeusia (loss of taste), meningitis, encephalitis, encephalopathy, and persistent fatigue. Nonetheless, it is unclear whether these symptoms are due to the direct viral tropism of brain cells or the result of systemic inflammation.

To explore whether the observed pathological symptoms in the central nervous system (CNS) are due to the direct involvement of SARS-CoV-2, several studies assessed viral tropism in a selection of brain cells. Due to the lack of accessibility to brain cells, these studies mostly relied on iPSC-derived cells and organoids [37, 45–47]. In one study, human iPSC-derived neural progenitor cells (NPCs) were reported to express known SARS-CoV-2 entry factors, such as ACE2, TMPRSS2, cathepsin L, and furin, and it was demonstrated that virions were able to replicate upon entry, where they reduced cell viability [45]. Nevertheless, this was not the case with brain organoids, where SARS-CoV-2 infections of NPCs were either absent or limited [37, 47]. Notably, Yi et al. (2020) reported that ACE2 expression was stable in the somas of mature neurons and rarely present in NPCs [48]. To assess neurotropism in a 3D multicellular environment that simulates a physical brain, viral infections have also been studied in neurospheres and cortical brain organoids [45]. Neurospheres exhibit the characteristics of early brain development, whereas cortical brain organoids mimic the physiological arrangements and development of the brain. In both neurospheres and cortical brain organoids, neurons were infected with SARS-CoV-2 viral particles upon exposure, albeit the number of infected cells was limited. Indeed, the ability of SARS-CoV-2 virions to infect neurons has been observed to be limited in other studies [37, 45–48]. It has been reported that spike-containing pseudovirions in dorsal forebrain organoids showed the ability to infect neurons with consistent ACE2 expression [48]. However, infectivity was not elevated in proportion to viral load and remained at 10% in neural cells, which implies that infectivity in the brain cortex is limited to a proportion of neural cells. Taken together, these studies showed that in the 3D context of brain organoids, SARS-CoV-2 virions do appear to show some degree of neurotropism, although the number of infected neurons might be limited in vivo. Nevertheless, even with a limited neurotroprim for neurons, it may still be enough to demonstrate the symptoms reported in COVID-19 patients. A limitation to the abovementioned studies is that they were not able to consider the penetration of SARS-CoV-2 through the blood–brain barrier (BBB), which would be necessary to model the impacts of an infected brain. The CNS is protected from the rest of the body by the BBB and the blood-CSF barrier (B-CSF-B), which prevent the entry of blood-borne pathogens such as viruses into the brain. The BBB separates the brain parenchyma from the systemic blood formed by tight junctions between endothelial cells that are tightly controlled by pericytes and glial cells [49]. On the other hand, B-CSF-B forms a barrier through a single layer of epithelial cells of the choroid plexus (ChP), which can be easily recapitulated with an organoid model in vitro. ChP organoid models can help elucidate how CSF is infected by SARS-CoV-2 virions [41].

A recently developed ChP model, which recapitulates the epithelial polarization of ChP cells, models the tight barrier that separates the surrounding cell culture media from the CSF-like fluid secreted by the ChP [47]. In the ChP organoids, SARS-CoV-2 virions were found to specifically target ChP cells, and when compared to cerebral organoids, they showed an inability to infect other types of brain cells (neurons or glia). These data suggest a specific neurotropism for the cells of the ChP. They also reported a complete absence of SARS-CoV-2 virions from neurons and other CNS cell types after infection unless infected with very large quantities of virions. This is in line with previous studies that showed limited neuronal infection and is in agreement with patient clinical data [40]. Furthermore, this study found that the virions target specific ChP populations that express lipoproteins, which might represent a more mature ChP stage [47]. Spike-containing pseudovirion infections were also found to disrupt the epithelial integrity of the ChP, disrupting its barrier function, in line with clinical data demonstrating leakage of blood proteins into the CSF in more than 40% of the studied patients [41, 47]. In another study that compared cortical, hippocampal, hypothalamic, and midbrain iPSC-derived organoids, they reported that SARS-CoV-2 most effectively targets hypothalamic organoids [46]. They also observed ChP epithelial cells in regions of some of the hippocampal organoids due to the proximity of the ChP to the hypothalamus during development. These ChP regions showed a greater density of infected cells. They also observed that infection of ChP organoids revealed a higher density of infected cells with increasing hours post-infection, indicating the ability of virions to replicate within these cells. Notably, these infections resulted in an increased rate of cell death, upregulation of inflammation-associated genes, and altered barrier/secretory function. Ultimately, these studies demonstrated that neurological COVID-19 symptoms may be due to an inflammatory response rather than direct infection of neurons. The limitations of studies with ChP organoids, however, are that these organoids lack vasculature and do not model the BBB.

A recent study integrated pericyte-like cells (PLCs), generated from iPSCs, into cortical brain organoids to form assembloids (3D fusions of multiple cell types) [50]. This enables the mimicry of a more realistic in vivo CNS environment. Pericytes are cells that are embedded within the basement membrane of endothelial cells, linking them to astrocytes [49]. They are especially important in regulating BBB permeability, inflammation, and neurodifferentiation. According to the mentioned report, SARS-CoV-2 can infect PLC-containing cortical organoids (PCCOs) and not cortical brain organoids, where they were found to infect both pericyte-like cells and astrocytes. Although SARS-CoV-2 has been previously reported to not infect astrocytes (as with neurons and microglia), unless, at high doses, the presence of PLCs might alter infectivity. This study also reported an increased apoptotic and inflammatory response in infected cells, suggesting their contribution to COVID-19 neurological symptoms.

#### Gastrointestinal system organoid

Although the lung is the most affected organ in individuals with COVID-19 infection, other organs, such as the intestines, kidneys, and liver, could also be involved. Of note, many individuals exhibit multisystem inflammation after COVID-19 recovery. Infection in the gastrointestinal system following SARS-CoV-2 causes disorders that commonly take place in severe cases compared to mild cases [51]. In fact, gastrointestinal system involvement in patients with COVID-19 was discovered after the detection of SARS-CoV-2 in stool samples of COVID-19 patients [3, 52]. In this regard, Wang et al. (2020) discovered the presence of SARS-CoV-2 RNA in stool specimens of 29% of COVID-19 patients [53]. Moreover, these researchers found a role for inflammatory cytokines released throughout SARS-CoV-2 infection on gastrointestinal system epithelial cells. SARS-CoV-2 isolation from COVID-19 patients’ stool samples using RT-qPCR suggested the potential of SARS-CoV-2 to cause enteric infection in COVID-19 patients [54].

Based on clinical reports, more than 50% of COVID-19 patients exhibit gastrointestinal symptoms, which are connected with more severe COVID-19 manifestations [55, 56]. Using colonic organoid models made of hPSCs, Han et al. (2021) discovered that 25% of patients who suffered from respiratory infection in their lungs also exhibited gastrointestinal complications, including diarrhea and vomiting. Of note, these groups of patients had worse COVID-19 outcomes [57]. Moreover, numerous reports have indicated that gastrointestinal symptoms of COVID-19 remain longer than the respiratory clearance of SARS-CoV-2. This phenomenon suggests that SARS-CoV-2 can switch to the gastrointestinal system after respiratory system infection. To examine this hypothesis, Giovanni et al. (2021) generated gastric organoids using human gastric stem cells derived from pediatric individuals with the purpose of providing a SARS-CoV-2 infection model. Based on the findings of this study, transcriptomic analysis of pediatric-derived gastric mini-organs could be more influenced by SARS-CoV-2, while the virus replication rate is lower in undifferentiated organoids than in adult organs. Moreover, the results of this study confirmed the potential of SARS-CoV-2 to transmit from the respiratory system to the gastrointestinal system [58].

Zang et al. (2020) reported that the intestine is a suitable place for SARS-CoV-2 replication and infection progression to other parts of the body. Similar to other studies, they confirmed the importance of ACE2 and TMPRSS2 as two essential internalization points for SARS-CoV-2 using human intestinal mini-organs. Furthermore, their results demonstrated that the higher expression of ACE2 on mature enterocytes can cause severe infection. An interesting finding was the effect of intestinal tract fluids on SARS-CoV-2 inactivation that was observed in the absence of SARS-CoV-2 in COVID-19-positive patients’ fecal samples [23]. Using hPSC-derived intestinal organoids, Han et al. (2021) reported that ACE2, which is expressed by various types of colonic cells, acts as an entry point for SARS-CoV-2. Moreover, physiologically relevant concentrations of SARS-CoV-2 entry inhibitors (such as imatinib, MPA, and quinacrine dihydrochloride (QNHC)) could significantly protect hPSC-derived colon organoids from SARS-CoV-2 infection. Such findings could serve as a drug-screening source for identifying the best drug candidate to manage the manifestations of COVID-19 [57].

Duan et al. (2020) created colonic mini-organs using hPSCs to investigate suitable medication for SARS-CoV-2 infection blockade. In their study, approximately 1280 FDA-approved antiviral drugs were investigated. Immunostaining and single-cell RNA-sequence analysis revealed that hPSC-derived colonic organoids express ACE2 and TMPRS2S2. These researchers compared the efficacy of three FDA-approved drugs for blocking virus entry into human cells via their receptors. These antiviral drugs included MPA, QNHC, and chloroquine. Based on their study findings, MPA and QNHC showed a significantly higher capacity for blocking virus entry (5 times higher) in comparison with chloroquine, which was applied to control SARS-CoV-2 infection in critical COVID-19 patients during the early days of the pandemic. Moreover, MPA is considered to be routinely used to block viral entry and replication [59].

As SARS-CoV-2 entrance requires TMPRSS and ACE, Krüger et al. (2021) created human intestinal organoids from pluripotent stem cells expressing TMPRSS and ACE to study the virus pathology and reaction to various drugs [62]. To date, remdesivir is known as an effective candidate for controlling SARS-CoV-2 infection. Remdesivir is an antiviral medication used for various types of viral infections. In fact, this medication was primarily industrialized for the treatment of hepatitis C and Ebola virus infections. Remdesivir has been approved for medical use as an emergency drug to manage SARS-CoV-2 infection in COVID-19 patients in many countries [60, 61]. Krüger et al. (2021) generated hPSC gastrointestinal organoid models to certify the efficiency of remdesivir in controlling the manifestation of SARS-CoV-2 infection in the gastrointestinal system. Immunostaining results for the SARS-CoV-2 entry receptor demonstrated high expression of ACE2 and TMPRSS in the gastrointestinal organoid model. This finding confirmed the potential of the organoids to be infected by SARS-CoV-2. Moreover, remdesivir showed an effective impact on SARS-CoV-2 infection inhibition even at low concentrations [62].

Additionally, famidine has been recommended as a medication for lowering disease severity in COVID-19 patients [63]. Famotidine is commonly used for the treatment of gastric disorders. This medication has been recommended for the reduction of inflammatory response side effects in COVID-19 patients [62]. Krüger et al. (2021) reported the inhibitory effects of remdesivir on SARS-CoV-2 replication in intestinal mini-organs. This finding showed that remdesivir can be considered a suitable medication for the management of gastrointestinal infection in COVID-19 patients. However, in contrast to previous studies, they did not observe any changes in SARS-CoV-2 replication capacity after the use of famotidine [62].

Another study by Duan et al. (2020) generated hPSC-derived colonic mini-organs to screen 1280 medications that were approved by the US FDA for the control of various types of viral infections. According to the RNA-seq results, QNHC and MPA were considered suitable candidates to block SARS-CoV-2 infection in the colonic organoid. QNHC is known as an antibiotic and antimalarial medication. In addition, MPA is a drug used to prevent organ rejection in kidney transplant recipients that is known to work on the immune system [59].

Almost 80% of human intestinal epithelial cells are intestinal absorptive cells called enterocytes. These cells are known as the main target for SARS-CoV-2 and have been used for the generation of intestinal organoids. Various studies have reported the presence of SARS-CoV-2 in the stool specimen of a COVID-19 patient who presented with diarrhea. Xiao et al. (2020) reported the presence of SARS-CoV-2 RNA in the stool specimens of 53% of COVID-19-positive patients (n: 73) [64]. Furthermore, Xu et al. (2020) detected SARS-CoV-2 RNA in stool specimens of COVID-19-positive pediatric cases [56]. In a further study, Lamers et al. (2020) reported the presence of viral particles using confocal microscopy and electron microscopy after infecting enterocytes in intestinal mini-organs (derived from human primary gut epithelial stem cells) with SARS-CoV-2. This finding showed the entry potential of SARS-CoV-2 into enterocytes. Differentiated enterocytes express high levels of ACE [65]. Therefore, intestinal epithelial cells provide an appropriate environment for SARS-CoV-2 replication. This finding confirms that intestinal organoids could be considered suitable models for studying COVID-19 [65].

Patients with severe COVID-19 are recognized by high production levels of proinflammatory and inflammatory cytokines, such as IL-1, IL-6 and TNF-α. Such cytokine profiles can cause immunity suppression and cytokine storms. This hyperinflammatory reaction could also lead to failure of multiple organs. Regarding the role of inflammatory cytokines (released throughout SARS-CoV-2 infection) in gut epithelial cells, Zhou et al. (2020) reported the upregulation of IFN-α, IFN-β and IFN-γ genes following SARS-CoV-2 intestinal infection. Furthermore, RT–qPCR analysis of intestinal mini-organs infected by SARS-CoV-2 showed human IFNL2 and IFNL3 upregulation [54]. Additionally, active SARS-CoV-2 replication and upregulation of IFN type III were detected following viral infections in human intestinal mini-organs in a study by Good et al. (2019) [66]. Recently, researchers have suggested that anti-inflammatory medications such as aspirin, ibuprofen, naproxen, and diclofenac can control hyperinflammatory reactions and cytokine storms in COVID-19 patients [67].

#### Liver organoid

Liver damage, which is detected via parenchymal liver enzymes, is frequently seen in patients with COVID-19. Remarkably, the mortality rate in COVID-19-positive cases who show liver dysfunction is very high. It would be extremely beneficial to identify virus tropism to set up plans for antiviral treatment. Creating organoids could be a promising method to achieve this goal. At the beginning of the COVID-19 pandemic, there were no reports of liver infection. Later, Ong et al. (2020) reported the presence of viral hepatitis in almost 50% of COVID-19-positive patients [68]. Consistent with these findings, Zhao et al. (2020) demonstrated the ability of SARS-CoV-2 to infect liver organoids. Their findings also presented increased cell mortality rates in these organoids [69].

The epithelial cells of the intrahepatic bile duct, called cholangiocytes, were found to be an ideal entry point for SARS-CoV-2. These cells could be mimicked in vitro using a human liver mini-organ system. Bile duct and liver cells both express ACE2, which makes them an ideal target for SARS-CoV-2 infection. In comparison to liver cells, bile duct epithelial cells express ACE2 at higher levels. This phenotype gives them the potential to play a crucial role in SARS-CoV-2 infection and immune responses [24, 70, 71]. Chai et al. (2020) confirmed this hypothesis by detecting the presence of SARS-CoV-2 in liver tissues through RT-PCR analysis [71]. Moreover, Wang et al. (2020) further confirmed this finding by detecting the presence of SARS-CoV-2 viral particles in the hepatocyte cytoplasm of COVID-19-positive cases [53].

Indeed, the underlying mechanism of liver injury in SARS-CoV-2 infection is still unclear. Chai et al. (2020) suggested that drug cytotoxicity, inflammatory responses, and direct viral infection could be responsible for the occurrence of liver injury in SARS-CoV-2-infected patients [71]. Using hPSC-derived liver organoids, Liu et al. (2020) demonstrated increased chemokine secretion following transcript profiling. They found that even though ACE2 is the main target for SARS-CoV-2, liver organoid cells show low susceptibility to virus entry. These findings suggest the need for other factors, in addition to ACE2, for virus entry [70].

#### Kidney organoids

SARS-CoV-2 pathogenesis can cause multiple organ failure, which can include kidney failure and result in acute kidney injury (AKI) [65, 74–77]. Some studies have demonstrated the direct role of SARS-CoV-2 in AKI [78–81]. Furthermore, kidney cell injury could also occur following COVID-19 resolution [74]. Piñeiro et al. (2021) reported the occurrence of kidney cell injury in 21.4% of COVID-19 patients [74].

Since the COVID-19 pandemic started, many reports have been released regarding the occurrence of kidney damage in COVID-19 patients [82, 83]. The detection of SARS-CoV-2 mRNA in the urine samples of patients suggests the potential of SARS-CoV-2 replication in the urinary system [84–86]. Jitske et al. (2022) infected human-iPSC-derived kidney organoids with SARS-CoV-2 to evaluate the direct influence of the virus on the kidney without the interference of any medical treatment. They reported that the organoid had no immune cell infiltration, and the infection of the kidney cells was found to be independent of the immune response [77]. They also reported an enhancement of profibrotic (e.g., TGFβ, EGFR, WNT, NOTCH, Hedgehog, FGF, PDGFR, JAK-STAT and connective tissue growth factor (CTGF) signaling pathways) and proinflammatory signaling pathways in hiPSC-derived kidney organoids via single-cell RNA sequencing [77].

Chugh et al. (2021) suggested that the FGF and TGFβ signaling pathways could be responsible for fibrosis development, which has not been detected in other organoid models, such as the lung and intestine [87]. Furthermore, their results indicated that COVID-19 patients exhibit increased kidney cell fibrosis, which is a hallmark of chronic kidney disease (CKD) [88, 89].

Infection following SARS-CoV-2 was found to be inhibited by human recombinant soluble ACE2 (hrsACE2) [90]. Allison et al. (2020) created human embryonic stem cell (ESC)-derived kidney mini-organs with the purpose of evaluating the role of hrsACE2 in the inhibition of SARS-CoV-2 and ACE2 interactions. Their results demonstrated that hrsACE2 could directly decrease SARS-CoV-2 replication and control its infection ability. SARS-CoV-2 RNA was detected via single-cell RNA sequencing one day after infection, which shows the occurrence of virus replication. Furthermore, hrsACE2 might block the binding of SARS-CoV-2 to target cells and its subsequent internalization [90].

In fact, only a few drugs with acceptable efficacy have been discovered for the management of COVID-19. Indeed, remdesivir is known as the most promising treatment strategy for COVID-19 manifestation management, although it does not have any effect on the mortality rate [91]. In a study by Monteil et al. (2021), human stem cell-derived kidney organoids were used to examine the potential of remdesivir in combination with recombinant soluble ACE2 for the treatment of COVID-19. Based on the results of this study, this combinatorial treatment approach controlled COVID-19 by targeting ACE2-dependent virus entry and inhibiting SARS-CoV-2 RNA replication capacity into host cells [91].

#### Retinal organoids

The retina is another organ in the human body affected by SARS-CoV-2. This organ shows the expression of ACE2 and TMPRSS2 in the inner parts and on the surface of the eye [92–94]. Various studies have reported SARS-CoV-2 RNA and spike protein detection in the retina cells of COVID-19 patients through single-cell RNA sequencing and immunohistochemical analysis, respectively. However, other studies failed to detect the virus from retina biopsies. These findings support the hypothesis that SARS-CoV-2 can infect retinal cells even without replication [95–100].

Garcia et al. (2020) generated human-iPSC-derived retina mini-organs expressing TMPRSS2 and ACE2 receptors to investigate whether the retina is susceptible to SARS-CoV-2 infection. Their analysis of immunofluorescence staining and mRNA confirmed the infection of retina organoids by SARS-CoV-2 through ACE2 and TMPRSS2. Based on their RNA-seq outcomes, the expression of inflammatory response genes, which are responsible for COVID-19 progression in retina organoids infected by SARS-CoV-2, was higher than that in the uninfected control groups. Moreover, they demonstrated that SARS-CoV-2 could act as an apoptosis activator in these cells [101].

In fact, the effect of SARS-CoV-2 on cell death is governed through the upregulation of genes associated with the endothelial cell apoptotic process (e.g., CCL2, THBS1, AQP1, and SERPINE1) [46]. In a study by Mulyadi Lai et al. (2021), researchers explained SARS-CoV-2 pathogenesis, which could be remarkably beneficial for drug discovery and development purposes [102]. In another study, Makovoz et al. (2020) analyzed hPSC-derived eye mini-organs via RNA sequencing. They evaluated ACE2 and TMPRSS2 expression levels in eye organoids. Based on their demonstration, in comparison to the central cornea, limbus cells show higher expression of ACE2 and TMPRSS2, putting them at a higher risk of SARS-CoV-2 infection. Moreover, they found that SARS-CoV-2 effectively suppresses the IFNβ response, which is consistent with infection transmission to other regions of the body [94]. Mulyadi Lai et al. (2021) created hPSC-derived retinal organoids to study retinal infection by SARS-CoV-2. The fact that SARS-CoV-2 can proliferate in different retina lineages encouraged researchers to hypothesize that retina organoids might be infected by SARS-CoV-2 [102]. Infection of retina organoids by SARS-CoV-2 causes induction of the expression of some inflammatory genes (such as interleukin 33, which is associated with retinal degeneration). Moreover, they reported a significant reduction in SARS-CoV-2 infection in retina organoids after using antibodies to block ACE2 receptors [102]. These researchers demonstrated the dependency of SARS-CoV-2 on ACE-2 for infecting retinal cells. Taking everything into account, the involvement of the retina in COVID-19 can be considered a safety alarm for monitoring retinal disorders following SARS-CoV-2 infection [103].

### SARS-CoV-2 variants

During genome replication viruses such as SARS-CoV-2, face genetic mutations or viral recombination that result in the creation of different variants from the original virus. SARS CoV 2 variants are classified based on their infectivity potential to public health and escaping potential from the host immune reaction, by WHO and other international healthcare associations around the world as Variants of Interest (VOI), Variants of Concern (VOC), and Variant Being Monitored (VBM). Numerous studies showed the usefulness of organoid models in the evaluation of SARS-CoV-2 variants infectivity, such as using human airway, alveolar and intestinal organoid models to study the Alpha variant (B.1.1.7) [104] and nasal epithelium-derived organoid culture system to show high infectivity potential of the Omicron and Delta variants than in comparison with other VOC. [105]. Several mutations in SARS-CoV-2 variants target the S1 domain of the S protein, in addition to those already occurring in the RBD, which cause the affinity enhancement of the virus to the human ACE2 receptor and as a result, increase the transmissibility of the virus. The function of these mutations is through neutralizing antibodies suppression. They stop neutralizing antibodies to target the receptor binding motif (RBM) in addition to the Receptor-Binding Domain (RBD) of the spike protein [106–109]. Alpha (B.1.1.7) [110], Beta (B.1.351) [111], Delta (B.1.617.2) [112] Gama (P.1) and Omicron (B.1.1.529), are the most common VOC. These variants have the most transmission potential and cause higher severity. Omicron (B.1.529) is the most recent variant [113, 114] which is more infectious than others. Based on the research the vaccines that induce neutralizing antibodies cannot effectively inhibit the Omicron variant [115–117]. The major SARS CoV 2 public health concern variants (VOC) and their information such as the number of mutations, country of their appearance for the first time, risk of their infection, vaccination efficacy, and potential therapeutic strategies are briefly explained in Table 1.

**Table 1:** The major SARS-CoV-2 variants

WHO label	Omicron [118]	Delta [119]	Alpha [110]	Beta [120]	Gamma [121]
Pango Lineage Code	B.1.1.529	B.1.617.2	B.1.1.7	B.1.351	P.1
First documented	November 2021	May 2021	September 2020	May 2020	November 2020
Country/Region	South Africa	India	United Kingdom	South Africa	Brazil
Number of Mutations	32 mutations on the spike protein	High	Approximately 23	High	High
Infectivity as compare to earlier variants	High	Twice more transmissible	1.5 times more transmissible	50% more transmissible	Estimated 1.7 to 2.4 times more transmissible
Symptoms	Mild headaches for several days (preliminary indications of similar symptoms as other variants)	Mild headaches, fever, persistent cough, sore throat (Shares symptoms as the original virus)	Fever, shortness of breath, cough, fatigue, change of sense of taste/smell (shares symptoms as the original virus)	Mild headaches, fever, persistent cough, sore throat (Shares symptoms as the original virus)	Mild headaches, fever, persistent cough, sore throat (Shares symptoms as the original virus)
Risk of Reinfection	High	High	High	High	High
Vaccination Efficacy	mRNA- based [122] Adenovirus-based [123] Recombinant protein-based [124] Inactivated virus-based [125]				
Potential Therapeutic Strategy	Blocking VOC entry	Targeting spike proteins via S1 Receptor-Binding Domain (RBD) affecting (e.g. Alpha variant) [126, 127] Using single-domains neutralizing antibodies against any of those targets such as Soluble human recombinant ACE2 (e.g., Alpha variant) [110] in kidney organoid model) [120] and human airway organoid model [121] Anti-RBD nanobodies isolated from llamas to neutralize RBD variants (All VOC) [122] Targeting host cell transmembrane protease serine 2 (TMPRSS2) by TMPRSS2 inhibitors (e.g., Camostat) [123], or A disintegrin and metalloprotease 17 (ADAM17) inhibitors [124]			
	Interrupting VOC replication	RNA dependent RNA polymerase inhibitors (e.g., Remdesivir, GS-441524) (e.g., Alpha variant) [125] Cas13d-based Prophylactic Antiviral CRISPR Human Cells (PAC-MAN) strategy that target reserved regions such as nucleocapsid protein and RdRp in SARS-CoV-2 viral genome [126]			

## Conclusion

**Fig. 2:**
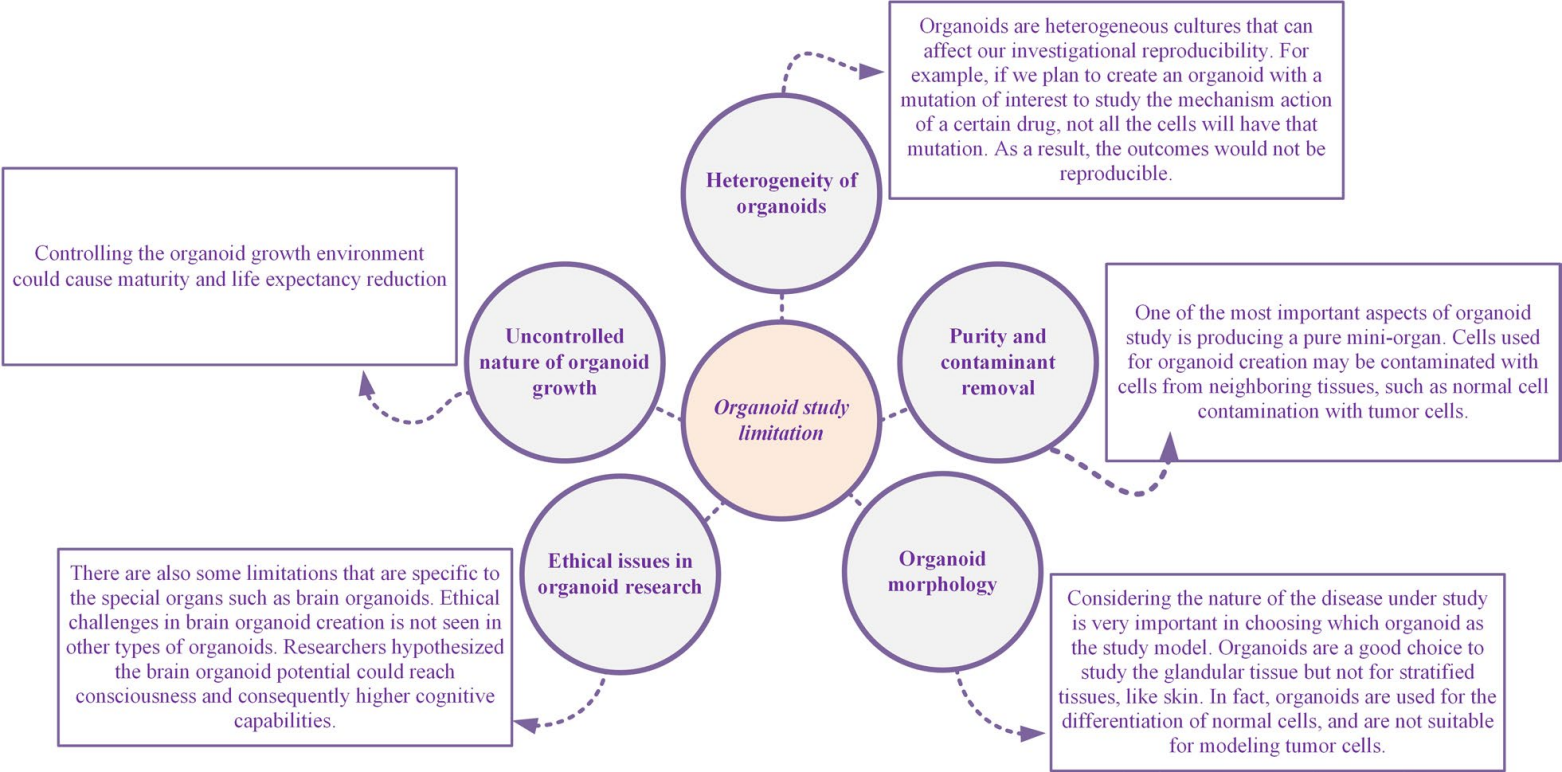
Organoid-based study limitations

The appearance of disease pandemics such as COVID-19 provides an urgent requirement for treatment discovery and progression. In fact, the organoid study model has demonstrated its potential in different aspects of SARS-CoV-2 investigation. Although organoid models are considered one of the best accessible methods to study SARS-CoV-2 biology and pathogenicity, this technique still has some limitations (Fig. 2). Technologies such as genome editing techniques could improve organoid models for disease modeling to discover and provide an effective therapeutic approach. Mimicking complex organs using the organoid strategy will improve our knowledge about controlling various types of diseases and improving personalized medicine. Furthermore, improvement in data analysis, troubleshooting and overcoming the challenges in mini-organ handling would provide a bright future for this approach of disease modeling and drug screening and discovery. Figure 2 shows the limitations in organoid-based studies.

## Data Availability

Not applicable.

## Abbreviations

COVID-19: Coronavirus disease-2019
SARS-CoV-2: Severe acute respiratory syndrome coronavirus 2
PSCs: Pluripotent stem cells
iPSCs: Induced pluripotent stem cells
ESCs: Embryonic stem cells
ASCs: Adult stem cells
ACE2: Angiotensin-converting enzyme 2
TMPRSS2: Transmembrane protease serine subfamily 2
MSCs: Mesenchymal stem cells
ARDS: Acute respiratory distress syndrome
hPSCs: Human pluripotent stem cells
MPA: Mycophenolic acid
TNF-alpha: Necrosis factor-alpha
AT2: Type II alveolar epithelial cell
GFP: Green fluorescent protein
CSF: Cerebrospinal fluid
CNS: Central nervous system
NPCs: Neural progenitor cells
BBB: Blood-brain barrier
B-CSF-B: Blood-CSF barrier
ChP: Choroid plexus
PLCs: Pericyte-like cells
PCCOs: PLC-containing cortical organoids
QNHC: Quinacrine dihydrochloride
AKI: Acute kidney injury
CTGF: Connective tissue growth factor
CKD: Chronic kidney disease
hrsACE2: Human recombinant soluble ACE2
ESC: Embryonic stem cell
